# Rational Design of π-Conjugated Tricoordinated Organoboron Derivatives With Thermally Activated Delayed Fluorescent Properties for Application in Organic Light-Emitting Diodes

**DOI:** 10.3389/fchem.2020.577834

**Published:** 2020-09-30

**Authors:** Ruifa Jin, Jingfan Xin

**Affiliations:** ^1^College of Chemistry and Life Sciences, Chifeng University, Chifeng, China; ^2^Inner Mongolia Key Laboratory of Photoelectric Functional Materials, Chifeng University, Chifeng, China

**Keywords:** tricoordinated organoboron derivatives, thermally activated delayed fluorescent (TADF), photophysical properties, reorganization energy, organic light-emitting diodes (OLEDs)

## Abstract

A series of donor–acceptor (D–A) tricoordinated organoboron derivatives (**1**–**10**) have been systematically investigated for thermally activated delayed fluorescent (TADF)-based organic light-emitting diode (OLED) materials. The calculated results show that the designed molecules exhibit small singlet-triplet energy gap (Δ*E*_ST_) values. Density functional theory (DFT) analysis indicated that the designed molecules display an efficient separation between donor and acceptor fragments because of a small overlap between donor and acceptor fragments on HOMOs and LUMOs. Furthermore, the delayed fluorescence emission color can be tuned effectively by introduction of different polycyclic aromatic fragments in parent molecule **1**. The calculated results show that molecules **2**, **3**, and **4** possess more significant Stokes shifts and red emission with small Δ*E*_ST_ values. Nevertheless, other molecules exhibit green (**1**, **7**, and **8**), light green (**6** and **10**), and blue (**5** and **9**) emissions. Meanwhile, they are potential ambipolar charge transport materials except that **4** and **10** can serve as electron and hole transport materials only, respectively. Therefore, we proposed a rational way for the design of efficient TADF materials as well as charge transport materials for OLEDs simultaneously.

## Introduction

Organic light-emitting diodes (OLEDs) have drawn considerable attention for applications in displaying and lighting fields owing to their outstanding advantages nowadays (Choy et al., 2014; Zhang et al., 2015; Im et al., 2017a; Liu et al., 2017b, 2018; Pal et al., 2018; Zhu et al., 2018). Unfortunately, their commercialization applications are still limited by the low device performance at high luminance and low external quantum efficiency (EQE) of the emitters. It is noteworthy that charge recombination results in 25% singlet and 75% triplet excitons in the traditional fluorescence process (Tang and Vanslyke, 1987; Burroughes et al., 1990). The triplet excitons cannot be directly utilized by emitters because they are spin forbidden and decay is non-emissive in producing undesired heat. As a consequence, the internal quantum efficiency (IQE) of OLEDs does not exceed 25% using singlet excitons for traditional fluorescent materials. Remarkably, it is challenging to develop highly efficient emitters utilizing triplet excitons. The phosphorescent emitters incorporating heavy metals can reach an IQE of nearly 100% by enhancing electron spin–orbit coupling (Baldo et al., 1998; Adachi et al., 2001; Xia et al., 2014). However, the applications of phosphorescent materials containing heavy metals are limited owing to their high cost and environmental contamination (Li et al., 2013; Liu et al., 2017a; Wang et al., 2018). Accordingly, it is critically important to develop novel metal-free materials, which can be functionalized as efficient emitters and exhibit environment friendliness. To address this issue, thermally activated delayed fluorescence (TADF) materials have been considered recently as promising candidates because of their potential in achieving 100% IQEs by harvesting all the triplet excitons (Uoyama et al., 2012; Wu et al., 2017; Yang et al., 2017; Chatterjee and Wong, 2019; Wang et al., 2019). As for TADF materials, a small energy gap (Δ*E*_ST_) between the lowest singlet (S_1_) and triplet (T_1_) states promotes efficient spin conversion from the triplet to singlet manifold by thermal activation through reverse intersystem crossing (RISC), subsequently resulting in fluorescence from the converted S_1_ to the ground (S_0_) states (Evans et al., 2018; Li et al., 2018b; Zhu et al., 2019). It is noteworthy that a small Δ*E*_ST_ is favorable for the RISC process and may obtain TADF. Furthermore, it is noticeable that the interplay between theory and experiment is capable of providing a deeper insight into the understanding of the optical and electronic properties of molecules in ground as well as excited states. Many theoretical research efforts have been made in this regard. It is critically important to get the relationship between topologic structure and optical as well as electronic properties for designing novel materials with improved properties. Density functional theory (DFT) and time-dependent DFT (TD-DFT) approaches have been remarkably successful in accurately evaluating a variety of ground and excited-state properties, in particular for TADF materials (Lu et al., 2015a,b; Wang et al., 2017; Hussain et al., 2019). In this regard, the rational design of a twisted donor–acceptor (D–A)-type structure can endow the molecules with TADF characteristics. These systems can efficiently reduce Δ*E*_ST_ in virtue of their separating the highest occupied molecular orbitals (HOMOs) and the lowest unoccupied molecular orbitals (LUMOs) (Geng et al., 2017; Li et al., 2018a). Additionally, D–A architectures are beneficial for the intramolecular charge transfer (ICT) in the excited states (Santos et al., 2016; Deng et al., 2019; Zhang et al., 2020). In addition, TADF materials with different donor moieties such as 9,9-dimethyl-9,10-dihydroacridine, 10H-phenoxazine, 5,10-dihydrophenazine, 10H-phenothiazine, 9H-carbazole, 3,6-di-tert-butyl-9H-carbazole, 9,9-diphenyl-9,10-dihydroacridine 9H-spiro[4,5]fluorene-9,10-dihydroacridine, 9-(3-(9H-carbazol-9-yl)phenyl)-9H-carbazole, and diphenylamine possess excellent electroluminescence performance (Bezvikonnyi et al., 2019; Hosokai et al., 2019; Jin et al., 2019; Joo et al., 2019; Kim et al., 2019; Sharma et al., 2019; Zhong et al., 2019). Meanwhile, there are many TADF materials with different acceptor moieties such as benzonitrile, triazines, sulfones, benzophenone, quinoxaline, and naphthalimide reported in literature (Li et al., 2016; Nobuyasu et al., 2016; Tsujimoto et al., 2017; Sommer et al., 2018; Wu et al., 2018; Yu et al., 2018). The HOMO/LUMO distribution and energy levels of the typical donor and acceptor moieties have been found in the relevant literature (Im et al., 2017b). Among the numerous reported TADF materials, organoboron compounds have drawn significant interest recently owing to their appealing optical properties in OLEDs. Because of the lack of electrons in the P_z_ orbital, the central boron atoms in the organoboron compounds exhibit strong electron-accepting ability through p–π conjugations, which is favorable for the ICT (Ji et al., 2017; Giustra and Liu, 2018; Liang et al., 2018; Meng et al., 2019).

**Scheme 1:**
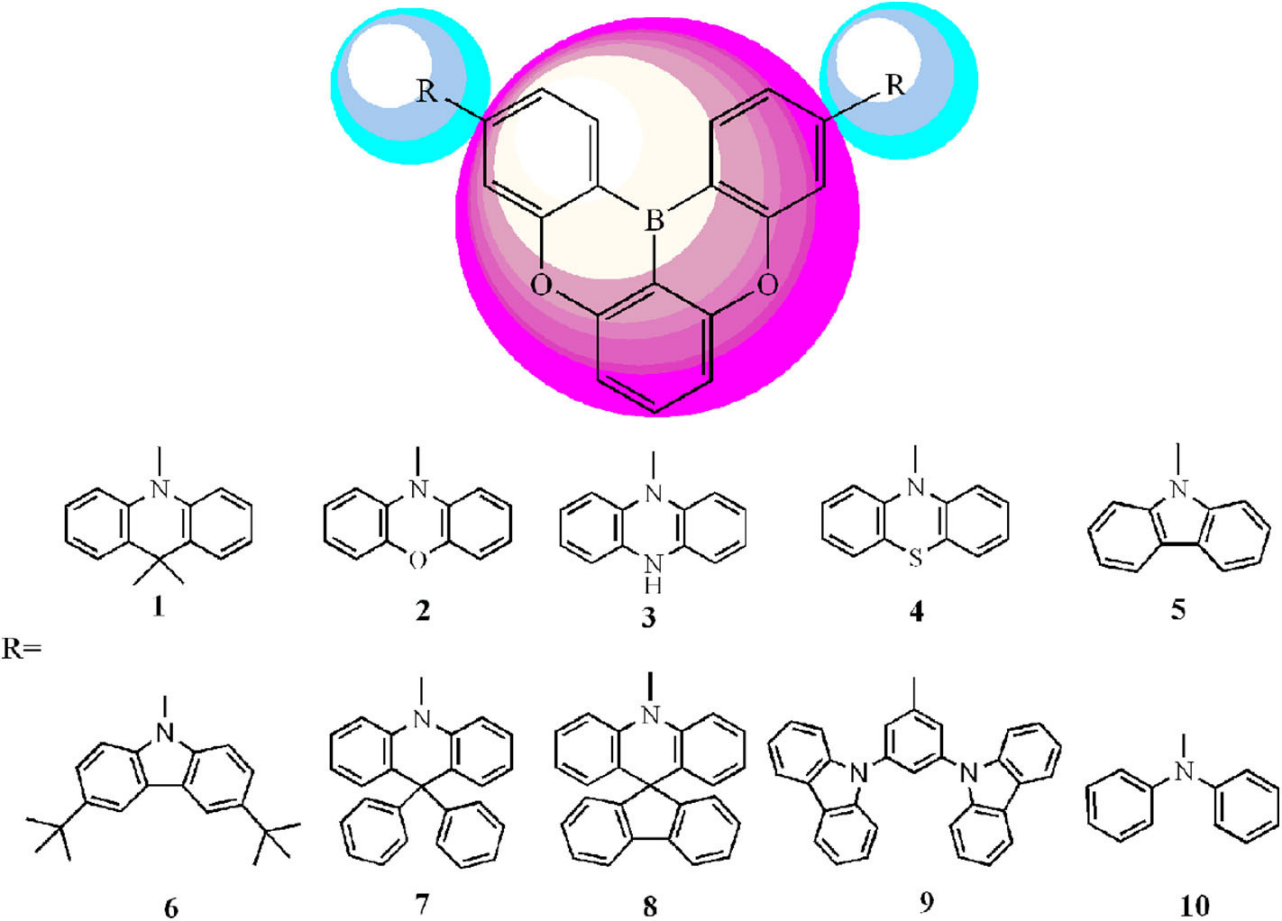
Molecule models of **1**–**10** investigated in this work.

Considering the merits and characteristics mentioned above, in this work, we design a series of novel D–A tricoordinated organoboron compounds with 5,9-dioxa-13b-boranaphtho[3,2,1-de]anthracene (DOBA) as electron acceptors and different polycyclic aromatic fragments as electron donors for TADF molecules (Scheme 1). Applying DFT and TD-DFT approaches, optoelectronic properties including frontier molecular orbital (FMO) energies (*E*_HOMO_ and *E*_LUMO_), HOMO–LUMO gaps (*E*_g_), adiabatic ionization potentials (*AIP*), adiabatic electron affinities (*AEA*), reorganization energy (λ), and Δ*E*_ST_ were systematically investigated. The absorption, fluorescence, and phosphorescence spectra of the designed molecules were predicted using the TD-DFT method. This provides a useful insight for designing novel TADF materials as well as charge transport materials for OLEDs.

## Computational Details

**Figure 1:**
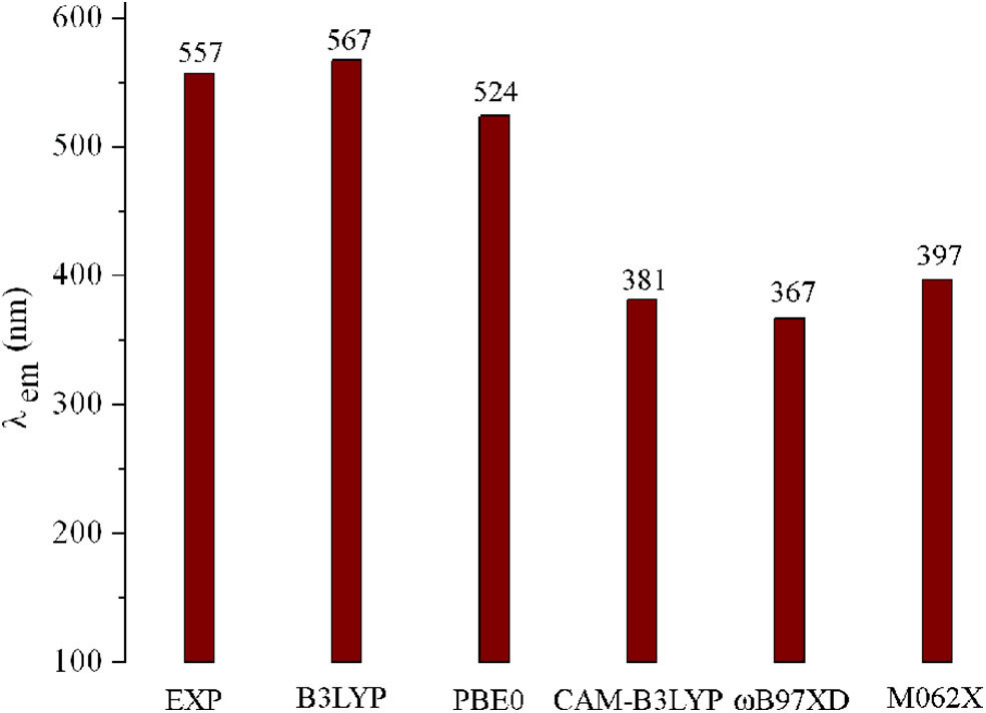
Calculated fluorescence wavelengths (λ_fl_) of **1** using various functionals, along with available experimental data.

Geometry optimizations for all the designed molecules in the S_0_ states were accomplished using the DFT method in the Gaussian 09 package (Frisch et al., 2009). The corresponding frequency calculations were carried out at the same level to prove the nature of each stationary point. Using the TD-DFT approach, the structures in S_1_ and T_1_ states were optimized. Under the optimized structures in S_0_, S_1_, and T_1_ states, their absorption, fluorescence, and phosphorescence spectra were predicted by using the TD-DFT method. Meanwhile, the Δ*E*_ST_ values were evaluated by adiabatic excitation energy. The 6-31G (d,p) basis set was selected for all the calculations in this work. With the aim to select reasonable exchange correlation functionals, six functionals including B3LYP (Becke, 1993), PBE0 (Adamo and Barone, 1999), ωB97XD (Chai and Head-Gordon, 2008), M062X (Zhao and Truhlar, 2008), and CAM-B3LYP (Yanai et al., 2004) were employed to optimize the geometries of molecule **1** in S_0_, S_1_, and T_1_ states. On the basis of the optimized geometries in S_0_ and S_1_ states, the absorption and fluorescence spectra were predicted using the corresponding TD-DFT method. The Δ*E*_ST_ values were also calculated using the different methods. The corresponding results as well as the experimental data are shown in Figure 1 and Supplementary Table 1. As visualized in Figure 1, obviously, the fluorescence emission wavelength (λ_fl_) obtained using the B3LYP functional (567 nm) is quite close to the experimental value (557 nm) (Meng et al., 2019), with the deviation of 10 nm. Furthermore, both the Δ*E*_ST_ values of molecule **1** obtained using B3LYP and PBE0 functionals (0.0068 and 0.0107 eV) are close to the experimental value (0.0091 eV). Additionally, comparing the optimized geometries of **1** (for the atom numbering, see Supplementary Figure 1) in S_0_ states with its crystal structure data [CCDC 1887610], one can find that the main geometrical parameters obtained using the B3LYP/6-31G(d,p) method are in better agreement with crystal structure data than those obtained with other methods (Supplementary Table 2). Therefore, geometry optimizations in S_0_, S_1_, and T_1_ states, band gaps *E*_g_, Δ*E*_ST_, absorption, fluorescence, and phosphorescence spectra of molecules under investigation were performed by the B3LYP/6-31G(d,p) and TD-B3LYP/6-31G(d,p) methods.

The reorganization energy λ is composed of external and internal reorganization energies (λ_ext_ and λ_int_). We focus only on the λ_int_ because λ_ext_ is quite complicated to predict at this stage. λ_int_ can be expressed as (Köse et al., 2007; Sancho-García, 2007):

(1) $$\begin{array}{l} \lambda =\lambda_{1}+\lambda_{2}=\left(E_{\pm }^{*}-E_{\pm }\right)+\left(E^{*}-E\right) \end{array}$$

Here, *E*_±_ and *E* stand for the energies of the charged and neutral states in the ground states, respectively; $E_{\pm }^{*}$ corresponds to the energy of the charged state with the optimized neutral molecule structure. E^*^ represents the energy of neutral molecule with the optimized charged geometry. Furthermore, *AIP* and *AEA* were calculated by the adiabatic potential-energy surfaces of neutral/charged species. *AIP* can be obtained with the energy difference between cation and neutral specie. *AEA* was determined by the energy difference between neutral and anion specie. The *AIP, AEA*, and λ for the electron (λ_e_) and hole (λ_h_) of the designed molecules were predicted at the B3LYP/6-31G(d,p) level.

## Results and Discussion

### Molecular Geometries in the Ground and Excited States

The main geometrical parameters of the designed molecules in S_0_, S_1_, and T_1_ states are given in Supplementary Table 3. The results displayed in Supplementary Table 3 reveal that there are no significant changes in the bond lengths of acceptor DOBA fragments for the designed molecules in S_0_ states. Compared with the parent molecule **1**, the bond length change mainly appears on the bonds which connect the DOBA acceptor and different donor fragments. Obviously, the lengths of C_5_-N_22_ bonds for molecules **2**, **3**, **5**–**8**, and **10** have been decreased by 0.005, 0.003, 0.02, 0.022, 0.001, and 0.024 Å, while the corresponding values of C_5_-N_22_ for **4** and C_5_-C_22_ for **9** have lengthened by 0.002 and 0.05 Å with respect to that of molecules **1**, respectively. Similar phenomena are found for C_18_-N_24_ bonds for **2**–**8** and **10** and C_18_-C_24_ bond for **9**. Furthermore, the designed molecules display a large twist angle (β) between their acceptor and donor fragments in the S_0_ states owing to their large steric hindrance. The β values of **1**–**4**, **7**, and **8** are about 81–100°, while the corresponding values of **5**, **6**, 9, and **10** are about 34–51°, respectively. The large β values facilitate for disrupting the electronic communication between D and A fragments. Comparing the geometrical parameters of the designed molecules in S_1_ states with those in the S_0_ states, the bond lengths of B_1_−C_2_, C_4_−C_5_, C_6_−C_7_, C_9_−C_10_, C_13_−C_14_, B_1_−C_15_, C_17_−C_18_, and C_19_−C_20_ (0.002–0.025 Å) are shortened, while other bond lengths are enlarged (0.001–0.025 Å), respectively. The more obvious bond length variations are found for the connecting bonds between acceptor and donor fragments for the designed molecules. The C_5_−N_12_ and C_18_−N_24_ bonds for **1**–**8** and **10** are stretched (0.005–0.038 Å and 0.005–0.038 Å) in the S_1_ states as compared to those in the S_0_ states, respectively. However, compared with the C_5_−C_12_ and C_18_−C_24_ bonds for **9** in the S_0_ state, obviously, they are shortened by 0.036 and 0.003 Å in the S_1_ state, respectively. Additionally, it can be seen that the twist angle β is closer to 90° in the S_1_ state as compared to those in the S_0_ states for the designed molecules except for **9**, whose two β values in the S_1_ state are smaller than those in S_0_ states, respectively. Apparently, inspection of Supplementary Table 3 reveals that the geometrical parameters in T_1_ states are similar to those in S_1_ states for **1**–**4**, **7**, and **8**. On the contrary, the molecular geometries in T_1_ states for **5**, **6**, **9**, and **10** are closer to those in S_0_ states, respectively.

### Frontier Molecular Orbitals

It is worth noting that the FMO energies (*E*_HOMO_ and *E*_LUMO_) and *E*_g_ play dominant roles for the optical and electronic properties. The molecular geometry is affected by electron density redistribution that is caused by an electronic excitation (Forés et al., 1999; Helal et al., 2011). The distributions of HOMO and LUMO in S_0_ states for the designed molecules are plotted in Supplementary Figure 2. Additionally, we investigated the contributions of A and D fragments to the FMOs and the overlap value (ρ) between D and A fragments on HOMOs and LUMOs, as displayed in Supplementary Table 4. An inspection in Supplementary Figure 2 reveals clearly that the HOMOs of **1**–**10** are mainly localized on the D fragments, whereas the corresponding LUMOs predominantly reside at the A fragments. The contributions of D fragments for HOMOs in **1**–**4** and **7**–**9** are 93.1, 94.4, 95.6, 95.4, 93.1, 92.8, and 99.4%, while the corresponding contributions for **5**, **6**, and **10** are 83.1, 85.8, and 66.5%, respectively. For the LUMOs, the contributions of A fragments in **1**–**8** are larger than 90%, while the corresponding contributions for **9** and **10** are 75.9 and 84.5%, respectively. Apparently, the HOMO to LUMO transitions lead the electronic density to flow from D to A fragments. The percentages of charge transfer are in the order of **4** (89.3%) > **3** (88.9%) > 2 (87.5%) > **7** (86.4%) > **8** (85.9%) > **1** (85.7%) > **6** (76.9%)> **9** (75.3%)> **5** (74.8%) > **10** (51%). Moreover, the overlap (ρ) between D and A fragments on HOMOs is in the range of 0.001–0.040, while the corresponding ρ values for LUMOs are 0.013–0.026, for the designed molecules except for **10**, whose ρ values on its HOMO and LUMO are 0.082 and 0.037, respectively. It indicates that the HOMO to LUMO transitions exhibit a strong charge transfer nature and slight electron exchange energy, resulting in small Δ*E*_ST_ values.

**Figure 2:**
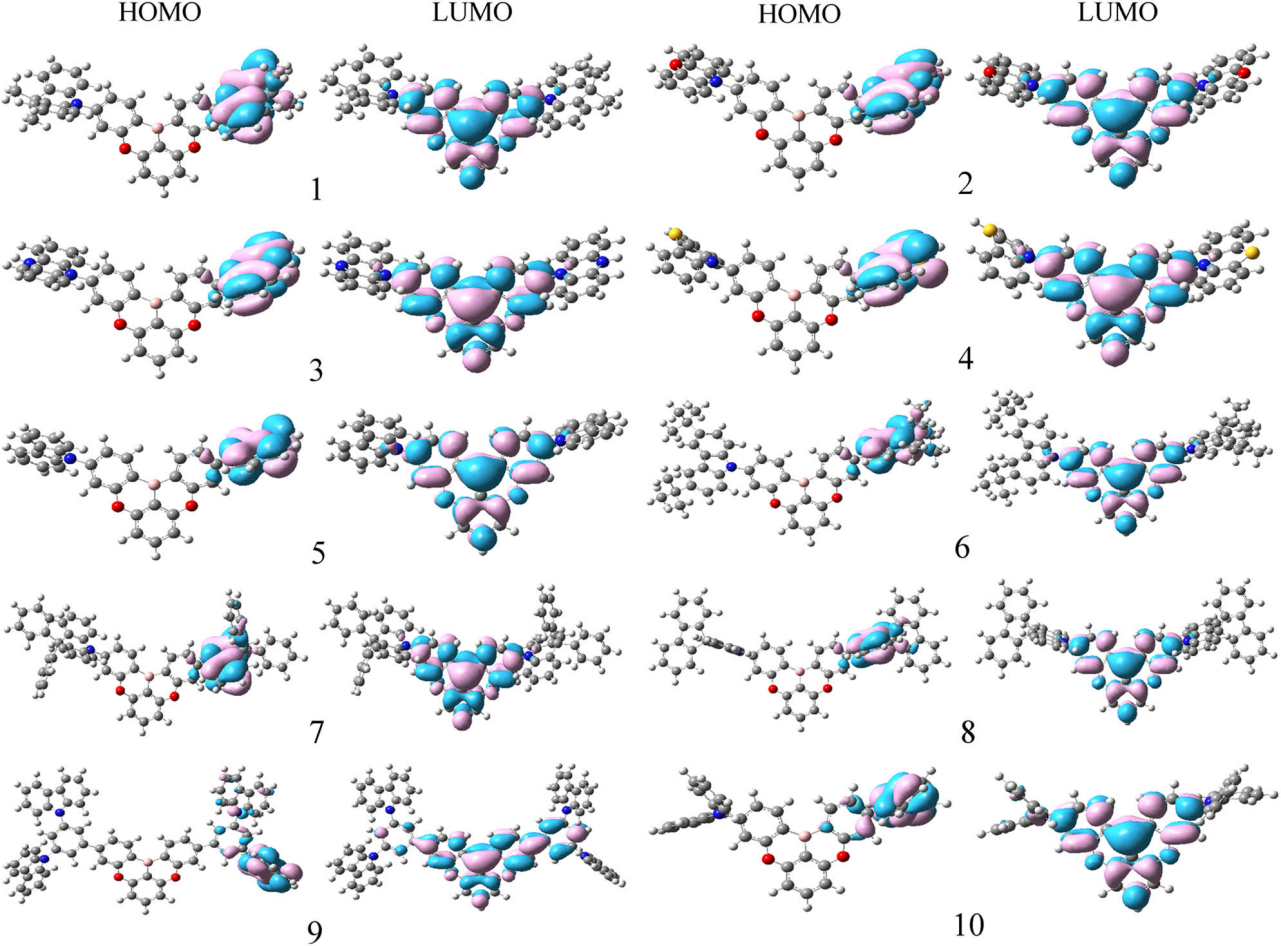
The distributions of HOMOs and LUMOs in S_1_ states for the designed molecules.

The qualitative HOMOs and LUMOs in S_1_ states for **1**–**10** are shown in Figure 2. The calculated *E*_HOMO_, *E*_LUMO_, and *E*_g_, the overlap (ρ) between D and A fragments, and the contributions of A and D fragments (in %) to the FMOs of **1**–**10** are summarized in Table 1. Comparing the HOMOs and LUMOs in S_1_ states with those in S_0_ states, we find that the electron density plots of both HOMOs and LUMOs in S_1_ states are similar to those in S_0_ states for all the investigated molecules, respectively. The HOMOs are mainly centralized on the D fragments, whereas the corresponding LUMOs predominantly distributed on the A fragments. The contributions of D fragments for HOMOs are about 91.2–99.9%. The contributions of A fragments for the LUMOs are about 90.5–93.8% except that the contribution of A fragment for **9** is 68.7%. The percentages of charge transfer from D to A fragments are in the order of **3** (89.5%) > **4** (89.1%) > 2 (88.6%) > **6** (88.1%) > **5** (87.8%) > **7** (87.3%) > **8** (86.6%)> **1** (86.3%)> **10** (81.7%) > **9** (68.6%). Clearly, the charge transfer character in S_1_ states is more significant than that in S_0_ states. Furthermore, the ρ values for HOMOs are in the range of 0.001–0.019, while the corresponding ρ values for LUMOs are about 0.014–0.037. It suggests that the HOMOs and LUMOs are also separated efficiently in the S_1_ state. This implies the efficient separation between D and A fragments, which display the potential TADF features. The sequence of *E*_HOMO_ and *E*_LUMO_ is **3** > **4** > **2** > **1**> **8** > **10** > **7** > **6** > **5** > **9** and **10** > **3** > **6** > **1**> **7** > **8** > **5** > **2** > **4** > **9**, respectively. Thus, the *E*_g_ values are in the order of **5** > **10** > **9** > **6**> **7** > **8** > **1** > **2** > **4** > **3**. It suggests that the *E*_g_ values of **2**–**4** decrease, while the corresponding value of **5**–**10** increases compared with that of **1**. Therefore, one can conclude that **2**–**4** may possess a red shift, whereas **5**–**10** could exhibit a blue shift in their emission spectra in comparison with the parent molecule **1**. It implies that the introduction of different aromatic heterocyclic donors can tune effectively the *E*_HOMO_, *E*_LUMO_, and *E*_g_ values for the designed molecules. Furthermore, we investigated the qualitative HOMO and LUMO plots in T_1_ states for **1**–**10** (Supplementary Figure 3). One can find that the qualitative HOMOs and LUMOs in T_1_ states are similar to those in S_1_ states for all the investigated molecules. It is also favorable for the RISC process from T_1_ to S_1_ states.

**Table 1:** The FMO energies *E*_HOMO_ and *E*_LUMO_, HOMO–LUMO gaps *E*_g_ (all in eV), HOMO and LUMO contributions (%), and the overlap between D and A fragments on HOMOs and LUMOs (ρ) of **1**–**10** in S_1_ states.

**Species**	**HOMO**				**LUMO**				***E*_*g*_**
	***E*_**HOMO**_**	**A**	**D**	**ρ**	***E*_**LUMO**_**	**A**	**D**	**ρ**	
**1**	−4.829	6.5	93.5	0.009	−2.146	92.8	7.2	0.017	2.683
**2**	−4.521	4.9	95.1	0.011	−2.269	93.5	6.5	0.014	2.253
**3**	−3.974	3.9	96.1	0.008	−2.115	93.4	6.6	0.015	1.858
**4**	−4.494	4.0	96.0	0.007	−2.275	93.1	6.9	0.016	2.219
**5**	−5.321	6.0	94.0	0.013	−2.196	93.8	6.2	0.014	3.125
**6**	−5.080	5.5	94.5	0.011	−2.129	93.6	6.4	0.014	2.952
**7**	−4.946	5.9	94.1	0.011	−2.159	93.2	6.8	0.016	2.787
**8**	−4.912	6.5	93.5	0.010	−2.195	93.1	6.9	0.018	2.717
**9**	−5.369	0.1	99.9	0.001	−2.394	68.7	31.3	0.037	2.976
**10**	−4.914	8.8	91.2	0.019	−1.918	90.5	9.5	0.021	2.996

*A, electron acceptors fragments; D, electron donors fragments*.

### Singlet-Triplet Energy Gap

Table 2 collected the vertical excitation energy (*E*_S1_ and *E*_T1_) in S_1_ and T_1_ states and Δ*E*_ST_ of **1**–**10** at the TD-B3LYP/6-31G (d,p) level. It is noticeable that the lower the Δ*E*_ST_ values, the easier the RISC process from the T_1_ to S_1_ states. The *E*_S1_ and *E*_T1_ values are in the orders **5** > **9** > **10** > **6** > **7** > **8** > **1** > **2** > **4** > **3** and **5** > **9** > **6** > **10** > **7** > **8** > **1** > **2** > **4** > **3**, respectively. As expected, the order of *E*_S1_ values is similar to those *E*_g_ values except that a sequential interchange is found between **9** and **10**. Moreover, the sequence of *E*_T1_ values is similar to *E*_S1_ values except that a sequential interchange is found between **6** and **10**. One can find that all the designed molecules possess small Δ*E*_ST_ values. The prediction of Δ*E*_ST_ values is in the sequence **10** (0.2659) > **9** (0.2600) > **5** (0.1257) > **6** (0.0618) > **7** (0.0078) > **8** (0.0069) > **1** (0.0068) > **2** (0.0065) > **4** (0.0062) > **3** (0.0057). The results displayed in Table 2 reveal that the Δ*E*_ST_ values of **2**–**4**, **7**, and **8** (0.0057–0.0078 eV) are similar to that for the parent molecule **1** (0.0068 eV). On the contrary, molecules **5, 6, 9**, and **10** have larger Δ*E*_ST_ values than the parent molecule **1**, especially for molecules **9** and **10**, whose Δ*E*_ST_ values are 0.2600 and 0.2659 eV, respectively. The reason might be that **2**–**4**, **7**, and **8** own the more effective separation between their donor/acceptor compositions (%) of the FMOs in the S_1_ state than those larger than 90% (Table 1 and Figure 2). Additionally, β is closer to 90° in the S_1_ state as compared to those of **5, 6, 9**, and **10**. For molecules **5** and **6**, among their two β, β values are both close to 90°, while another β values are −60.6 and 59.1°, respectively. However, molecule **6** has two electron donor tert-butyl groups in donor fragments, which is more favorable for charge transfer and in turn decreases the Δ*E*_ST_ value. As a consequence, the Δ*E*_ST_ value of **6** is smaller than that of **5**. For molecules **9** and **10**, their smaller two β values may decrease the effective separation between their donor and acceptor fragments. Two β values are −12.7 and 32.4° for **9** and −51.7 and 77.2° for **10** in S_1_ states. It indicates that the introduction of 10H-phenoxazine (**2**) 5,10-dihydrophenazine (**3**), 10H-phenothiazine (**4**), 9,9-diphenyl-9,10-dihydroacridine (**7**), and 9H-spiro[4,5]fluorene-9,10-dihydroacridine (**8**) donor fragments does not significantly affect the Δ*E*_ST_ values compared with the parent molecule with 9,9-dimethyl-9,10-dihydroacridine donor fragments (**1**). On the contrary, the introduction of 9H-carbazole (**5**), 3,6-di-tert-butyl-9H-carbazole (**6**), 9-(3-(9H-carbazol-9-yl)phenyl)-9H-carbazole (**9**), diphenylamine (**10**), and donor fragments increase the Δ*E*_ST_ values compared with parent molecule **1**. As a consequence, the RISC rate constant (*k*_RISC_) of **1**–**4**, **7**, and **8** should be higher than those of molecules **5, 6, 9**, and **10** because a small Δ*E*_ST_ value is beneficial for the high *k*_RISC_ value.

**Table 2:** The vertical excitation energy (*E*_S1_ and *E*_T1_) and singlet triplet energy gap (Δ*E*_ST_) of **1**–**10** at the TD-B3LYP/6-31G(d,p) level (in eV).

**Species**	***E*_**S1**_**	***E*_**T1**_**	****Δ***E*_**ST**_**
**1**	2.186	2.180	0.0068
**2**	1.773	1.768	0.0065
**3**	1.707	1.702	0.0057
**4**	1.763	1.758	0.0062
**5**	2.643	2.645	0.1257
**6**	2.480	2.552	0.0618
**7**	2.290	2.285	0.0078
**8**	2.222	2.216	0.0069
**9**	2.556	2.556	0.2600
**10**	2.510	2.452	0.2659
Exp			0.0091

*Exp, experimental results of **1** were taken from Meng et al. (2019)*.

### Photophysical Properties

The calculated wavelength of delayed fluorescence emission (λ_TADF_), phosphorescence emission (λ_ph_), absorption (λ_abs_), and Stokes shift of **1**–**10** are listed in Table 3. As visualized in Figure 2 and Supplementary Figures 2, **3**, the FMO distributions in S_0_, S_1_, and T_1_ states possess π characteristics. The results presented in Table 3 show that the λ_abs_ values of **2**–**10** have slight bathochromic shifts compared with that of the parent compound **1**, respectively. The λ_TADF_ values of **1**–**10** follow the tendency **3** > **4** > **2** > **1** > **8** > **7** > **6** > **10** > **9** > **5**, which is similar to the reverse order of *E*_g_ values. By comparing with parent molecule **1**, the λ_TADF_ values of **2**–**4** exhibit bathochromic shifts, 132.2, 159.5, and 136.9 nm, respectively, due to the increased electron-donating ability of the donor fragments. Conversely, it can be noted that the λ_TADF_ values of **5**–**10** have hypsochromic shifts, 97.9, 67.2, 25.7, 9.1, 82, and 73.1 nm compared with that of **1**, respectively. Obviously, **2**–**4** possess more significant Stokes shifts (326.6, 320.0, and 330.5 nm) and red emission with small Δ*E*_ST_ values (0.0065, 0.0057, and 0.0062 eV). Nevertheless, **1**, **7**, and **8** exhibit green emissions with Stokes shifts, 196.7, 169.8, and 183.1 nm, respectively. In addition, **6** and **10** show light green emissions, while **5** and **9** display blue emissions with small Stokes shifts. The Stokes shifts of **5**, **6**, **9**, and **10** are 60.0, 75.0, 68.1, and 92.2 nm, respectively. Accordingly, the delayed fluorescence emission color can be tuned effectively by introduction of different polycyclic aromatic fragments as electron donors in the parent molecule. This implies that the designed molecules are expected to be the promising candidates for TADF materials, particularly for **2**–**4**, **7**, and **8**. Furthermore, the λ_ph_ values are similar to the λ_TADF_ values. The small difference between the λ_TADF_ and λ_ph_ values should facilitate for achieving the TADF phenomenon.

**Table 3:** The delayed fluorescence emission wavelength (λ_TADF_) and phosphorescence emission wavelength (λ_ph_), corresponding to the absorption wavelength (λ_abs_), and Stokes shift of **1**–**10** at the TD-B3LYP/6-31G (d,p) level.

**Species**	****λ_abs_****	****λ_TADF_****	****λ_ph_****	**Stokes shift**
**1**	370.4	567.1	568.6	196.7
**2**	372.7	699.3	701.4	326.6
**3**	406.6	726.6	728.7	320.0
**4**	372.6	703.1	705.2	330.5
**5**	409.2	469.2	468.7	60.0
**6**	424.9	499.9	485.8	75.0
**7**	371.6	541.4	542.7	169.8
**8**	374.9	558.0	559.5	183.1
**9**	417.0	485.1	485.2	68.1
**10**	401.7	494.0	505.7	92.2
Exp	386	557	533	171

*Exp, experimental results of **1** were taken from Meng et al. (2019)*.

### Charge Transport Properties

It is quite clear that the charge injection and charge transfer play dominant roles in the device performance of OLEDs. λ can be used to estimate the charge transfer rate. The *AIP* and *AEA* are used to evaluate the energy barrier for the injection of holes and electrons. Generally, the low λ value corresponds to the big charge transfer rate (Marcus, 1964, 1993). The smaller the *AIP* value and the larger the *AEA* value, the easier the injection of holes and electrons, respectively. The calculated λ_e_, λ_h_, *AIP*, and *AEA* of **1**–**10** are listed in Table 4. As presented in Table 4, we find that **3** and **5** have the smallest and largest *AIP* values (5.151 and 6.387 eV), respectively. The *AIP* values of **1**–**10** are observed in the following: **5** > **9** > **6** > **7** > **10** > **8** > **1** > **4** > **2** > **3**, suggesting that the hole injection and transportations of **2**–**4** are expected to be easier than the others with respect to parent molecule **1**. On the other hand, the *AEA* values of **3**, **5**, **6**, and **10** are lower, while the corresponding values of **2**, **4**, and **7**–**9** are higher than that of **1** (0.878 eV). The order of the *AEA* of **1**–**10** is as follows: **9** > **4** > **2** > **8** > **7** > **1** > **5** > **3** > **6** > **10**; this implies that the abilities to accept electrons in **2**, **4**, and **7**–**9** are improved with respect to parent molecule **1**. So, we can deduce that the introduction of 10H-phenoxazine (**2**) and 10H-phenothiazine (**4**) donor fragments will enhance both the hole and electron injection abilities, whereas the introduction of 9,9-diphenyl-9,10-dihydroacridine (**7**), 9H-spiro[4,5]fluorene-9,10-dihydroacridine (**8**), and 9-(3-(9H-carbazol-9-yl)phenyl)-9H-carbazole (**9**) donor fragments can improve the electron injection ability only compared with that of parent molecule **1**.

**Table 4:** Calculated molecular λ_e_, λ_h_, *AIP*, and *AEA* (all in eV) of **1**–**10** at the B3LYP/6-31G(d,p) level.

**Species**	*****λ***_**h**_**	*****λ***_**e**_**	***AIP***	***AEA***
**1**	0.204	0.135	5.858	0.878
**2**	0.115	0.144	5.681	0.991
**3**	0.215	0.136	5.151	0.831
**4**	0.390	0.140	5.832	1.000
**5**	0.051	0.183	6.387	0.868
**6**	0.061	0.192	6.086	0.822
**7**	0.120	0.151	5.968	0.906
**8**	0.067	0.134	5.877	0.968
**9**	0.033	0.249	6.196	1.235
**10**	0.072	0.383	5.947	0.546

For charge transport materials in OLEDs, generally, TPD (λ_h_ = 0.290 eV) and Alq3 (λ_e_ = 0.276 eV) are used as typical hole and electron transport materials, respectively (Gruhn et al., 2002; Lin et al., 2005). According to Table 4, the calculated λ_h_ values of **1**–**3** and **5**–**10** are smaller than those of TPD. It suggests that the hole transfer rates of the designed molecules except for **4** may be higher than that of TPD. The λ_h_ values are in the order **4** > **3** > **1** > **2** > **7** > **10** > **8** > **6** > **5** > **9**. On the other hand, the λ_e_ values of **1**–**9** are smaller than that of Alq3. It implies that the electron transfer rates of **1**–**9** may be higher than that of Alq3, suggesting that their electron transfer rates might be lower than that of Alq3. The order of the predicted λ_e_ is as follows: **10** > **9** > **6** > **5** > **7** > **2** > **3** > **6** > **1** > **8**. Remarkably, the difference between λ_h_ and λ_e_ for **1**–**3**, **5**–**9** (0.029–0.216 eV) is small enough, suggesting that they are potential ambipolar charge transport materials. Nevertheless, **4** and **10** can serve as electron and hole transport materials only, respectively.

## Conclusion

In summary, a series of novel D–A tricoordinated organoboron derivatives have been systematically investigated for TADF-based OLED materials. The calculated results show that the designed molecules exhibit small Δ*E*_ST_ values, suggesting that they can be used as excellent TADF candidates. DFT analysis indicated that the designed molecules display efficient separation between donor and acceptor fragments because of the small overlap between donor and acceptor fragments (ρ) on HOMOs and LUMOs. Furthermore, the delayed fluorescence emission color can be tuned effectively by introduction of different polycyclic aromatic fragments in the parent molecule. Molecules **2**, **3**, and **4** possess more significant Stokes shifts (326.6, 320.0, and 330.5 nm) and red emission with small Δ*E*_ST_ values (0.0065, 0.0057, and 0.0062 eV). Nevertheless, other molecules exhibit green (**1**, **7**, and **8**), light green (**6** and **10**), and blue (**5** and **9**) emissions. Meanwhile, they are potential good ambipolar charge transport materials except that **4** and **10** can serve as electron and hole transport materials only, respectively. Therefore, we proposed a rational way for the design of efficient TADF materials as well as charge transport materials for OLEDs simultaneously.

## Data Availability Statement

The original contributions presented in the study are included in the article/Supplementary Material, further inquiries can be directed to the corresponding author/s.

## Author Contributions

RJ conceived and designed the research, and headed, wrote, and revised the manuscript. JX contributed to the performance and analysis of the frontier molecular orbitals, absorption fluorescence, and phosphorescence spectra, and the reorganization energies. Both authors contributed to the manuscript revision and read and approved the submitted version.

## Conflict of Interest

The authors declare that the research was conducted in the absence of any commercial or financial relationships that could be construed as a potential conflict of interest.
