# Supplementary material for: Rational Design of π-Conjugated Tricoordinated Organoboron Derivatives With Thermally Activated Delayed Fluorescent Properties for Application in Organic Light-Emitting Diodes
Source: Front Chem. 2020 Sep 30;8:577834. doi: 10.3389/fchem.2020.577834 (PMC7554541; doi:10.3389/fchem.2020.577834)
Supplement: Supplementary file 1 [file Table_1.DOC]

**Rational Design of π-Conjugated Tricoordinated Organoboron Derivatives with Thermally Activated Delayed Fluorescent Properties for Application in Organic Light-Emitting Diodes**

Ruifa Jin^1,2*^ and Jingfan Xin^1,2^

^1^College of Chemistry and Life Sciences, Chifeng University, Chifeng 024000, China

^2^Inner Mongolia Key Laboratory of Photoelectric Functional Materials, Chifeng University, Chifeng 024000, China

**Supplementary**

**List of Contents**

**Table S1** The calculated absorption and fluorescence wavelengths (λ_abs_ and λ_fl_, in nm) of **1** using different methods, along with available experimental data.

**Table S2** Calculated structural parameters like bond lengths and dihedral angles in the S_0_ optimized geometries using different functional as well as the crystal data.

**Table S3** Optimized bond lengths (Å) of **1**–**10** in the S_0_ and S_1_ states at B3LYP/6-31G(d,p) and TD-B3LYP/6-31G(d,p), respectively, and differences between the S_0_ and S_1_ States (Δ*L*_S1-S0_).

**Table S4** The HOMO and LUMO Contributions (%) and the overlap between D and A fragments on HOMOs and LUMOs (*ρ*) of **1**–**10** in S_0_ states at the B3LYP/6-31G (d,p) level.

**Figure S1** Geometry of **1** along with atom numbering.

**Figure S2** The distributions of HOMOs and LUMOs in S_0_ states for the designed molecules.

**Figure S3** The distributions of HOMOs and LUMOs in T_1_ states for the designed molecules.

**Table S1**

The calculated Δ*E*_ST_ values, absorption and fluorescence wavelengths (λ_abs_ and λ_fl_, in nm) of **1** using different methods, along with available experimental data.

| **1** | Δ*E*_ST_ | λ_abs_ | λ_fl_ |
| --- | --- | --- | --- |
| B3LYP | 0.0068 | 370 | 567 |
| PBE0 | 0.0107 | 357 | 524 |
| CAM-B3LYP | 0.5705 | 317 | 381 |
| *ω*B97XD | 0.6216 | 312 | 367 |
| M062X | 0.2324 | 317 | 397 |
| Exp | 0.0091 | 386 | 557 |

*Exp, Experimental results of* ***1*** *were taken from Ref. (*Meng et al.; 2019*).*

**Table S2**

Calculated structural parameters like bond lengths and dihedral angles in the S_0_ optimized geometries using different functional as well as the crystal data.

|  | B3LYP | PBE0 | CAM-B3LYP | M062X | *ω*B97XD | Crystal data |
| --- | --- | --- | --- | --- | --- | --- |
| R (1,2) | 1.551 | 1.548 | 1.548 | 1.550 | 1.549 | 1.549 |
| R (2,3) | 1.416 | 1.412 | 1.411 | 1.413 | 1.411 | 1.415 |
| R (3,4) | 1.386 | 1.382 | 1.379 | 1.380 | 1.380 | 1.374 |
| R (4,5) | 1.407 | 1.404 | 1.402 | 1.404 | 1.403 | 1.380 |
| R (5,6) | 1.386 | 1.382 | 1.379 | 1.380 | 1.380 | 1.373 |
| R (6,7) | 1.402 | 1.399 | 1.398 | 1.401 | 1.399 | 1.390 |
| R (7,8) | 1.371 | 1.362 | 1.365 | 1.364 | 1.363 | 1.378 |
| R (8,9) | 1.365 | 1.356 | 1.359 | 1.359 | 1.358 | 1.376 |
| R (9,10) | 1.394 | 1.390 | 1.388 | 1.390 | 1.390 | 1.382 |
| R (10,11) | 1.396 | 1.392 | 1.390 | 1.392 | 1.392 | 1.372 |
| R (11,12) | 1.396 | 1.392 | 1.390 | 1.392 | 1.392 | 1.388 |
| R (12,13) | 1.394 | 1.390 | 1.388 | 1.390 | 1.390 | 1.373 |
| R (13,14) | 1.407 | 1.403 | 1.400 | 1.402 | 1.401 | 1.399 |
| R (1,14) | 1.520 | 1.518 | 1.516 | 1.520 | 1.518 | 1.505 |
| R (1,15) | 1.551 | 1.548 | 1.548 | 1.550 | 1.549 | 1.555 |
| R (15,16) | 1.416 | 1.412 | 1.411 | 1.413 | 1.411 | 1.398 |
| R (16,17) | 1.386 | 1.382 | 1.379 | 1.380 | 1.380 | 1.370 |
| R (17,18) | 1.407 | 1.404 | 1.402 | 1.404 | 1.403 | 1.395 |
| R (18,19) | 1.386 | 1.382 | 1.379 | 1.380 | 1.380 | 1.365 |
| R (19,20) | 1.402 | 1.399 | 1.398 | 1.401 | 1.399 | 1.390 |
| R (20,21) | 1.371 | 1.362 | 1.365 | 1.364 | 1.363 | 1.376 |
| R (21,13) | 1.365 | 1.356 | 1.359 | 1.359 | 1.358 | 1.372 |
| R (5,22) | 1.435 | 1.427 | 1.430 | 1.430 | 1.428 | 1.443 |
| R (18,24) | 1.435 | 1.427 | 1.430 | 1.430 | 1.428 | 1.437 |
| β (4,5,22,23) | 80.9 | 79.2 | 79.3 | 74.6 | 81.2 | 87.9 |
| β (17,18,24,25) | 80.9 | 79.2 | 79.3 | 74.6 | 81.2 | 91.6 |

**Table S3**

Optimized bond lengths (Å) of **1**–**10** in the S_0_ and S_1_ states at B3LYP/6-31G(d,p) and TD-B3LYP/6-31G(d,p), respectively, and differences between the S_0_ and S_1_ states (Δ*L*_S1-S0_)

|  | **1** | | | |  | **2** | | | |
| --- | --- | --- | --- | --- | --- | --- | --- | --- | --- |
|  | S_0_ | S_1_ | T_1_ | Δ*L*_S1-S0_ |  | S_0_ | S_1_ | T_1_ | Δ*L*_S1-S0_ |
| R (1,2) | 1.551 | 1.541 | 1.541 | -0.01 |  | 1.551 | 1.542 | 1.541 | -0.009 |
| R (2,3) | 1.416 | 1.426 | 1.426 | 0.01 |  | 1.415 | 1.425 | 1.426 | 0.01 |
| R (3,4) | 1.386 | 1.392 | 1.392 | 0.006 |  | 1.386 | 1.392 | 1.392 | 0.006 |
| R (4,5) | 1.407 | 1.392 | 1.392 | -0.015 |  | 1.406 | 1.393 | 1.393 | -0.013 |
| R (5,6) | 1.386 | 1.405 | 1.406 | 0.019 |  | 1.388 | 1.405 | 1.406 | 0.017 |
| R (6,7) | 1.402 | 1.392 | 1.392 | -0.01 |  | 1.401 | 1.392 | 1.392 | -0.009 |
| R (7,8) | 1.371 | 1.372 | 1.372 | 0.001 |  | 1.371 | 1.372 | 1.372 | 0.001 |
| R (8,9) | 1.365 | 1.387 | 1.387 | 0.022 |  | 1.366 | 1.387 | 1.387 | 0.021 |
| R (9,10) | 1.394 | 1.390 | 1.390 | -0.004 |  | 1.393 | 1.390 | 1.390 | -0.003 |
| R (10,11) | 1.396 | 1.401 | 1.401 | 0.005 |  | 1.396 | 1.401 | 1.401 | 0.005 |
| R (11,12) | 1.396 | 1.397 | 1.397 | 0.001 |  | 1.396 | 1.397 | 1.397 | 0.001 |
| R (12,13) | 1.394 | 1.395 | 1.395 | 0.001 |  | 1.393 | 1.395 | 1.395 | 0.002 |
| R (13,14) | 1.407 | 1.404 | 1.404 | -0.003 |  | 1.407 | 1.404 | 1.404 | -0.003 |
| R (1,14) | 1.520 | 1.529 | 1.529 | 0.009 |  | 1.520 | 1.529 | 1.529 | 0.009 |
| R (1,15) | 1.551 | 1.544 | 1.545 | -0.007 |  | 1.551 | 1.543 | 1.543 | -0.008 |
| R (15,16) | 1.416 | 1.420 | 1.420 | 0.004 |  | 1.415 | 1.420 | 1.420 | 0.005 |
| R (16,17) | 1.386 | 1.391 | 1.391 | 0.005 |  | 1.386 | 1.391 | 1.391 | 0.005 |
| R (17,18) | 1.407 | 1.400 | 1.400 | -0.007 |  | 1.406 | 1.398 | 1.398 | -0.008 |
| R (18,19) | 1.386 | 1.397 | 1.397 | 0.011 |  | 1.389 | 1.400 | 1.400 | 0.011 |
| R (19,20) | 1.402 | 1.394 | 1.394 | -0.008 |  | 1.401 | 1.393 | 1.394 | -0.008 |
| R (20,21) | 1.371 | 1.381 | 1.381 | 0.01 |  | 1.371 | 1.381 | 1.381 | 0.01 |
| R (21,13) | 1.365 | 1.374 | 1.374 | 0.009 |  | 1.366 | 1.374 | 1.375 | 0.008 |
| R (5,22) | 1.435 | 1.464 | 1.463 | 0.029 |  | 1.430 | 1.461 | 1.460 | 0.031 |
| R (18,24) | 1.435 | 1.441 | 1.441 | 0.006 |  | 1.430 | 1.436 | 1.436 | 0.006 |
| β (4,5,22,23) | 80.9 | 90.1 | 90.1 | 9.2 |  | 94.7 | 90.2 | 90.2 | -4.5 |
| β (17,18,24,25) | 80.9 | 82.1 | 82.1 | 1.2 |  | 99.2 | 90.9 | 90.9 | -8.3 |
|  | **3** | | | |  | **4** | | | |
|  | S_0_ | S_1_ | T_1_ | Δ*L*_S1-S0_ |  | S_0_ | S_1_ | T_1_ | Δ*L*_S1-S0_ |
| R (1,2) | 1.551 | 1.543 | 1.544 | -0.008 |  | 1.551 | 1.543 | 1.542 | -0.008 |
| R (2,3) | 1.415 | 1.420 | 1.420 | 0.005 |  | 1.415 | 1.425 | 1.425 | 0.01 |
| R (3,4) | 1.386 | 1.391 | 1.391 | 0.005 |  | 1.386 | 1.393 | 1.393 | 0.007 |
| R (4,5) | 1.406 | 1.399 | 1.399 | -0.007 |  | 1.403 | 1.392 | 1.392 | -0.011 |
| R (5,6) | 1.387 | 1.399 | 1.399 | 0.012 |  | 1.391 | 1.405 | 1.405 | 0.014 |
| R (6,7) | 1.402 | 1.393 | 1.394 | -0.009 |  | 1.401 | 1.393 | 1.393 | -0.008 |
| R (7,8) | 1.371 | 1.382 | 1.382 | 0.011 |  | 1.372 | 1.372 | 1.372 | 0 |
| R (8,9) | 1.365 | 1.374 | 1.374 | 0.009 |  | 1.366 | 1.387 | 1.387 | 0.021 |
| R (9,10) | 1.394 | 1.395 | 1.395 | 0.001 |  | 1.394 | 1.390 | 1.390 | -0.004 |
| R (10,11) | 1.396 | 1.397 | 1.397 | 0.001 |  | 1.396 | 1.401 | 1.401 | 0.005 |
| R (11,12) | 1.396 | 1.401 | 1.401 | 0.005 |  | 1.396 | 1.397 | 1.397 | 0.001 |
| R (12,13) | 1.394 | 1.390 | 1.390 | -0.004 |  | 1.394 | 1.394 | 1.394 | 0 |
| R (13,14) | 1.407 | 1.402 | 1.402 | -0.005 |  | 1.407 | 1.404 | 1.404 | -0.003 |
| R (1,14) | 1.520 | 1.529 | 1.529 | 0.009 |  | 1.520 | 1.529 | 1.529 | 0.009 |
| R (1,15) | 1.551 | 1.542 | 1.542 | -0.009 |  | 1.551 | 1.542 | 1.543 | -0.009 |
| R (15,16) | 1.416 | 1.425 | 1.425 | 0.009 |  | 1.415 | 1.419 | 1.419 | 0.004 |
| R (16,17) | 1.386 | 1.392 | 1.392 | 0.006 |  | 1.386 | 1.391 | 1.391 | 0.005 |
| R (17,18) | 1.407 | 1.393 | 1.393 | -0.014 |  | 1.404 | 1.396 | 1.396 | -0.008 |
| R (18,19) | 1.387 | 1.405 | 1.405 | 0.018 |  | 1.390 | 1.402 | 1.402 | 0.012 |
| R (19,20) | 1.402 | 1.392 | 1.392 | -0.01 |  | 1.401 | 1.393 | 1.393 | -0.008 |
| R (20,21) | 1.371 | 1.373 | 1.373 | 0.002 |  | 1.372 | 1.382 | 1.382 | 0.01 |
| R (21,13) | 1.365 | 1.387 | 1.387 | 0.022 |  | 1.366 | 1.375 | 1.375 | 0.009 |
| R (5,22) | 1.432 | 1.437 | 1.437 | 0.005 |  | 1.437 | 1.466 | 1.465 | 0.029 |
| R (18,24) | 1.432 | 1.461 | 1.460 | 0.029 |  | 1.437 | 1.443 | 1.442 | 0.006 |
| β (4,5,22,23) | 81.2 | 85.0 | 85.0 | 3.8 |  | 96.2 | 90.3 | 90.3 | -5.9 |
| β (17,18,24,25) | 83.5 | 90.1 | 90.0 | 6.6 |  | 96.9 | 98.0 | 98.0 | 1.1 |
|  | **5** | | | |  | **6** | | | |
|  | S_0_ | S_1_ | T_1_ | Δ*L*_S1-S0_ |  | S_0_ | S_1_ | T_1_ | Δ*L*_S1-S0_ |
| R (1,2) | 1.549 | 1.545 | 1.536 | -0.004 |  | 1.549 | 1.540 | 1.536 | -0.009 |
| R (2,3) | 1.415 | 1.419 | 1.429 | 0.004 |  | 1.415 | 1.426 | 1.434 | 0.011 |
| R (3,4) | 1.384 | 1.391 | 1.382 | 0.007 |  | 1.384 | 1.391 | 1.383 | 0.007 |
| R (4,5) | 1.408 | 1.399 | 1.412 | -0.009 |  | 1.409 | 1.394 | 1.407 | -0.015 |
| R (5,6) | 1.391 | 1.400 | 1.408 | 0.009 |  | 1.392 | 1.407 | 1.416 | 0.015 |
| R (6,7) | 1.400 | 1.393 | 1.387 | -0.007 |  | 1.399 | 1.391 | 1.386 | -0.008 |
| R (7,8) | 1.372 | 1.381 | 1.376 | 0.009 |  | 1.372 | 1.374 | 1.375 | 0.002 |
| R (8,9) | 1.366 | 1.375 | 1.375 | 0.009 |  | 1.366 | 1.385 | 1.380 | 0.019 |
| R (9,10) | 1.394 | 1.395 | 1.395 | 0.001 |  | 1.394 | 1.390 | 1.391 | -0.004 |
| R (10,11) | 1.396 | 1.396 | 1.398 | 0 |  | 1.396 | 1.401 | 1.400 | 0.005 |
| R (11,12) | 1.396 | 1.401 | 1.397 | 0.005 |  | 1.396 | 1.397 | 1.396 | 0.001 |
| R (12,13) | 1.394 | 1.390 | 1.396 | -0.004 |  | 1.394 | 1.395 | 1.395 | 0.001 |
| R (13,14) | 1.406 | 1.402 | 1.400 | -0.004 |  | 1.406 | 1.404 | 1.403 | -0.002 |
| R (1,14) | 1.522 | 1.530 | 1.531 | 0.008 |  | 1.523 | 1.530 | 1.531 | 0.007 |
| R (1,15) | 1.549 | 1.540 | 1.538 | -0.009 |  | 1.549 | 1.545 | 1.543 | -0.004 |
| R (15,16) | 1.415 | 1.426 | 1.427 | 0.011 |  | 1.415 | 1.418 | 1.421 | 0.003 |
| R (16,17) | 1.385 | 1.391 | 1.383 | 0.006 |  | 1.384 | 1.391 | 1.387 | 0.007 |
| R (17,18) | 1.408 | 1.394 | 1.411 | -0.014 |  | 1.409 | 1.400 | 1.404 | -0.009 |
| R (18,19) | 1.392 | 1.407 | 1.405 | 0.015 |  | 1.392 | 1.400 | 1.401 | 0.008 |
| R (19,20) | 1.399 | 1.391 | 1.388 | -0.008 |  | 1.399 | 1.394 | 1.392 | -0.005 |
| R (20,21) | 1.372 | 1.373 | 1.376 | 0.001 |  | 1.372 | 1.381 | 1.377 | 0.009 |
| R (21,13) | 1.366 | 1.386 | 1.375 | 0.02 |  | 1.366 | 1.375 | 1.375 | 0.009 |
| R (5,22) | 1.415 | 1.427 | 1.415 | 0.012 |  | 1.413 | 1.451 | 1.421 | 0.038 |
| R (18,24) | 1.415 | 1.452 | 1.416 | 0.037 |  | 1.413 | 1.427 | 1.420 | 0.014 |
| β (4,5,22,23) | -51.1 | -60.6 | -51.3 | -9.5 |  | -49.6 | -90.0 | -52.5 | -40.4 |
| β (17,18,24,25) | 50.6 | 89.7 | 51.5 | 39.1 |  | 49.0 | 59.1 | 52.5 | 10.1 |
|  | **7** | | | |  | **8** | | | |
|  | S_0_ | S_1_ | T_1_ | Δ*L*_S1-S0_ |  | S_0_ | S_1_ | T_1_ | Δ*L*_S1-S0_ |
| R (1,2) | 1.552 | 1.541 | 1.540 | -0.011 |  | 1.552 | 1.544 | 1.544 | -0.008 |
| R (2,3) | 1.415 | 1.426 | 1.427 | 0.011 |  | 1.415 | 1.419 | 1.419 | 0.004 |
| R (3,4) | 1.386 | 1.392 | 1.392 | 0.006 |  | 1.386 | 1.391 | 1.391 | 0.005 |
| R (4,5) | 1.404 | 1.392 | 1.392 | -0.012 |  | 1.405 | 1.398 | 1.398 | -0.007 |
| R (5,6) | 1.390 | 1.407 | 1.407 | 0.017 |  | 1.388 | 1.399 | 1.399 | 0.011 |
| R (6,7) | 1.401 | 1.392 | 1.392 | -0.009 |  | 1.402 | 1.394 | 1.394 | -0.008 |
| R (7,8) | 1.372 | 1.373 | 1.373 | 0.001 |  | 1.371 | 1.381 | 1.381 | 0.01 |
| R (8,9) | 1.365 | 1.386 | 1.386 | 0.021 |  | 1.365 | 1.375 | 1.375 | 0.01 |
| R (9,10) | 1.394 | 1.390 | 1.390 | -0.004 |  | 1.394 | 1.395 | 1.395 | 0.001 |
| R (10,11) | 1.396 | 1.400 | 1.400 | 0.004 |  | 1.396 | 1.397 | 1.397 | 0.001 |
| R (11,12) | 1.396 | 1.397 | 1.397 | 0.001 |  | 1.396 | 1.401 | 1.401 | 0.005 |
| R (12,13) | 1.394 | 1.395 | 1.395 | 0.001 |  | 1.394 | 1.390 | 1.390 | -0.004 |
| R (13,14) | 1.407 | 1.404 | 1.404 | -0.003 |  | 1.407 | 1.402 | 1.402 | -0.005 |
| R (1,14) | 1.521 | 1.530 | 1.530 | 0.009 |  | 1.521 | 1.529 | 1.529 | 0.008 |
| R (1,15) | 1.552 | 1.546 | 1.546 | -0.006 |  | 1.552 | 1.540 | 1.540 | -0.012 |
| R (15,16) | 1.415 | 1.419 | 1.419 | 0.004 |  | 1.415 | 1.426 | 1.426 | 0.011 |
| R (16,17) | 1.386 | 1.391 | 1.391 | 0.005 |  | 1.386 | 1.392 | 1.392 | 0.006 |
| R (17,18) | 1.407 | 1.399 | 1.399 | -0.008 |  | 1.405 | 1.392 | 1.393 | -0.013 |
| R (18,19) | 1.387 | 1.397 | 1.397 | 0.01 |  | 1.388 | 1.405 | 1.406 | 0.017 |
| R (19,20) | 1.401 | 1.395 | 1.395 | -0.006 |  | 1.401 | 1.392 | 1.392 | -0.009 |
| R (20,21) | 1.371 | 1.381 | 1.381 | 0.01 |  | 1.371 | 1.373 | 1.373 | 0.002 |
| R (21,13) | 1.365 | 1.374 | 1.374 | 0.009 |  | 1.365 | 1.387 | 1.386 | 0.022 |
| R (5,22) | 1.434 | 1.463 | 1.462 | 0.029 |  | 1.434 | 1.440 | 1.440 | 0.006 |
| R (18,24) | 1.434 | 1.441 | 1.441 | 0.007 |  | 1.434 | 1.463 | 1.463 | 0.029 |
| β (4,5,22,23) | 97.0 | 92.3 | 91.6 | -4.7 |  | 87.1 | 89.8 | 89.7 | 2.7 |
| β (17,18,24,25) | 84.4 | 82.6 | 82.6 | -1.8 |  | 86.7 | 90.4 | 90.8 | 3.7 |
|  | **9** | | | |  | **10** | | | |
|  | S_0_ | S_1_ | T_1_ | Δ*L*_S1-S0_ |  | S_0_ | S_1_ | T_1_ | Δ*L*_S1-S0_ |
| R (1,2) | 1.550 | 1.525 | 1.551 | -0.025 |  | 1.546 | 1.541 | 1.533 | -0.005 |
| R (2,3) | 1.415 | 1.429 | 1.415 | 0.014 |  | 1.416 | 1.419 | 1.442 | 0.003 |
| R (3,4) | 1.384 | 1.377 | 1.385 | -0.007 |  | 1.382 | 1.390 | 1.377 | 0.008 |
| R (4,5) | 1.411 | 1.423 | 1.411 | 0.012 |  | 1.414 | 1.402 | 1.417 | -0.012 |
| R (5,6) | 1.394 | 1.421 | 1.395 | 0.027 |  | 1.397 | 1.402 | 1.428 | 0.005 |
| R (6,7) | 1.399 | 1.384 | 1.400 | -0.015 |  | 1.397 | 1.392 | 1.381 | -0.005 |
| R (7,8) | 1.372 | 1.384 | 1.373 | 0.012 |  | 1.374 | 1.382 | 1.376 | 0.008 |
| R (8,9) | 1.366 | 1.370 | 1.367 | 0.004 |  | 1.366 | 1.374 | 1.379 | 0.008 |
| R (9,10) | 1.394 | 1.395 | 1.396 | 0.001 |  | 1.395 | 1.394 | 1.392 | -0.001 |
| R (10,11) | 1.396 | 1.397 | 1.394 | 0.001 |  | 1.396 | 1.397 | 1.400 | 0.001 |
| R (11,12) | 1.396 | 1.396 | 1.399 | 0 |  | 1.396 | 1.401 | 1.395 | 0.005 |
| R (12,13) | 1.394 | 1.395 | 1.392 | 0.001 |  | 1.395 | 1.389 | 1.395 | -0.006 |
| R (13,14) | 1.407 | 1.403 | 1.406 | -0.004 |  | 1.406 | 1.403 | 1.402 | -0.003 |
| R (1,14) | 1.521 | 1.534 | 1.526 | 0.013 |  | 1.526 | 1.527 | 1.531 | 0.001 |
| R (1,15) | 1.550 | 1.555 | 1.535 | 0.005 |  | 1.546 | 1.545 | 1.541 | -0.001 |
| R (15,16) | 1.415 | 1.415 | 1.440 | 0 |  | 1.416 | 1.424 | 1.422 | 0.008 |
| R (16,17) | 1.384 | 1.387 | 1.366 | 0.003 |  | 1.382 | 1.394 | 1.384 | 0.012 |
| R (17,18) | 1.411 | 1.409 | 1.450 | -0.002 |  | 1.414 | 1.391 | 1.411 | -0.023 |
| R (18,19) | 1.394 | 1.400 | 1.445 | 0.006 |  | 1.397 | 1.406 | 1.405 | 0.009 |
| R (19,20) | 1.399 | 1.396 | 1.378 | -0.003 |  | 1.397 | 1.394 | 1.390 | -0.003 |
| R (20,21) | 1.372 | 1.378 | 1.378 | 0.006 |  | 1.374 | 1.371 | 1.377 | -0.003 |
| R (21,13) | 1.365 | 1.373 | 1.367 | 0.008 |  | 1.366 | 1.388 | 1.374 | 0.022 |
| R (5,22) | 1.485 | 1.450 | 1.485 | -0.035 |  | 1.411 | 1.431 | 1.413 | 0.02 |
| R (18,24) | 1.485 | 1.482 | 1.417 | -0.003 |  | 1.411 | 1.462 | 1.418 | 0.051 |
| β (4,5,22,23) | -34.2 | -12.9 | -34.2 | 21.3 |  | -42.1 | -51.7 | -42.1 | -9.6 |
| β (17,18,24,25) | 35.9 | 32.4 | 6.6 | -3.5 |  | 33.7 | 77.2 | 41.5 | 43.5 |

**Table S4** The HOMO and LUMO Contributions (%) and the overlap between D and A fragments on HOMOs and LUMOs (*ρ*) of **1**–**10** in S_0_ states at the B3LYP/6-31G (d,p) level.

| **Species** | HOMO | | |  | LUMO | | |
| --- | --- | --- | --- | --- | --- | --- | --- |
|  | A | D | *ρ* |  | A | D | *ρ* |
| **1** | 6.9 | 93.1 | 0.016 |  | 92.6 | 7.4 | 0.017 |
| **2** | 5.6 | 94.4 | 0.015 |  | 93.1 | 6.9 | 0.015 |
| **3** | 4.4 | 95.6 | 0.011 |  | 93.3 | 6.7 | 0.016 |
| **4** | 4.6 | 95.4 | 0.013 |  | 93.9 | 6.1 | 0.013 |
| **5** | 16.9 | 83.1 | 0.040 |  | 91.7 | 8.3 | 0.024 |
| **6** | 14.2 | 85.8 | 0.036 |  | 91.1 | 8.9 | 0.026 |
| **7** | 6.9 | 93.1 | 0.017 |  | 93.3 | 6.7 | 0.014 |
| **8** | 7.2 | 92.8 | 0.013 |  | 93.1 | 6.9 | 0.018 |
| **9** | 0.6 | 99.4 | 0.001 |  | 75.9 | 24.1 | 0.024 |
| **10** | 33.5 | 66.5 | 0.082 |  | 84.5 | 15.5 | 0.037 |

*A, electron acceptors fragments.*

*D, electron donors fragments.*


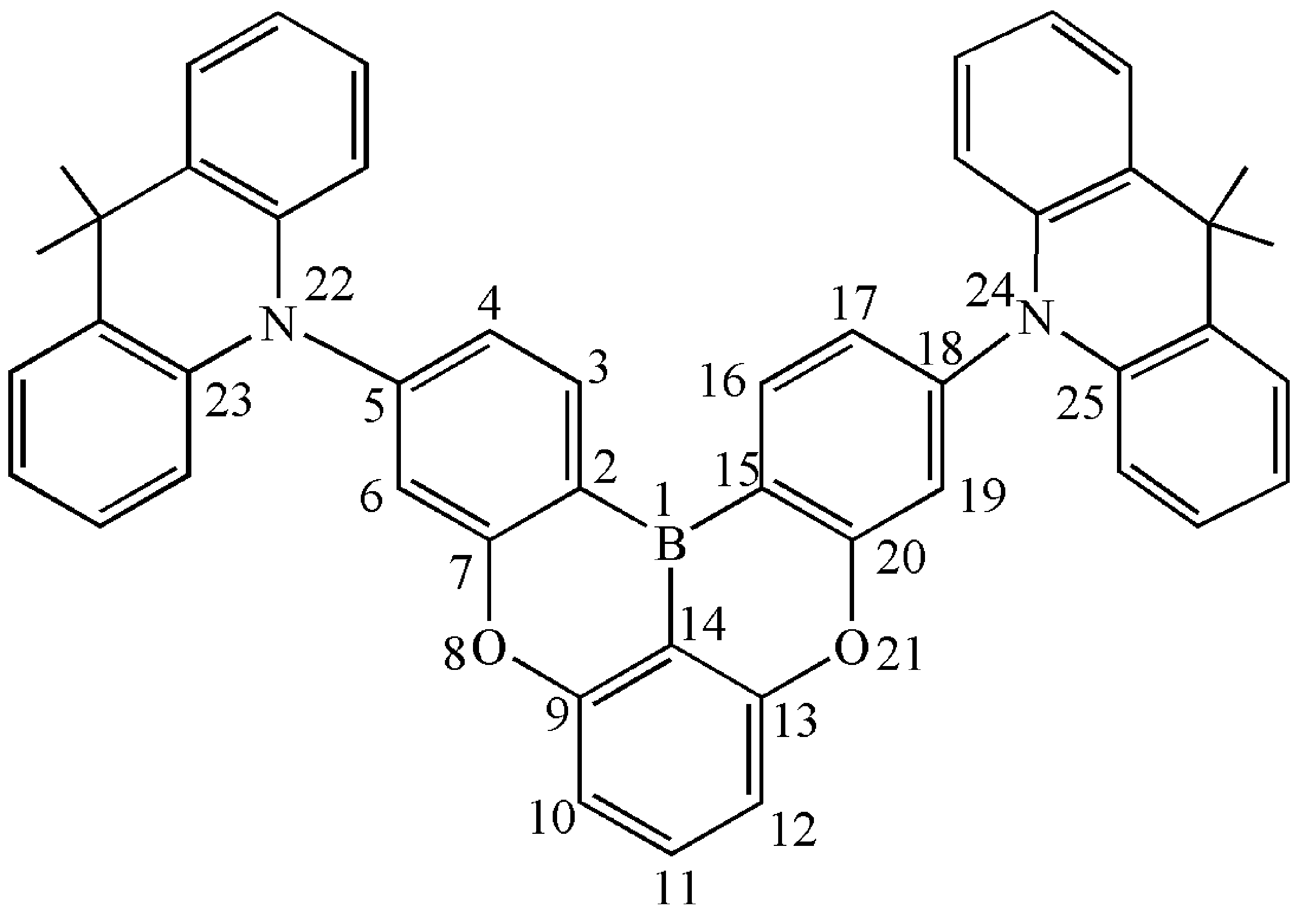


**Figure S1**. Geometry of **1** along with atom numbering.

**
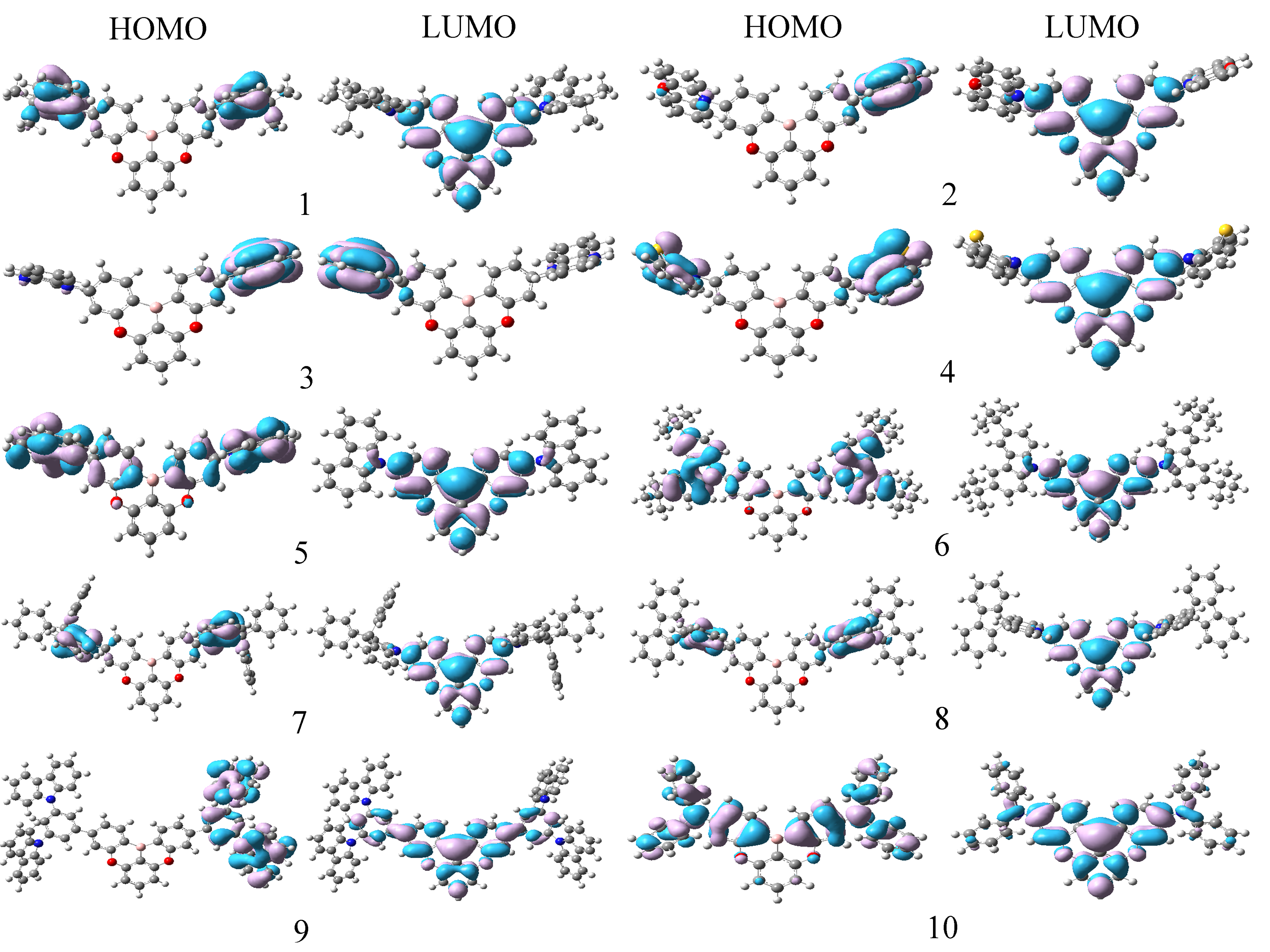
**

**Figure S2** The distributions of HOMOs and LUMOs in S_0_ states for the designed molecules.

**
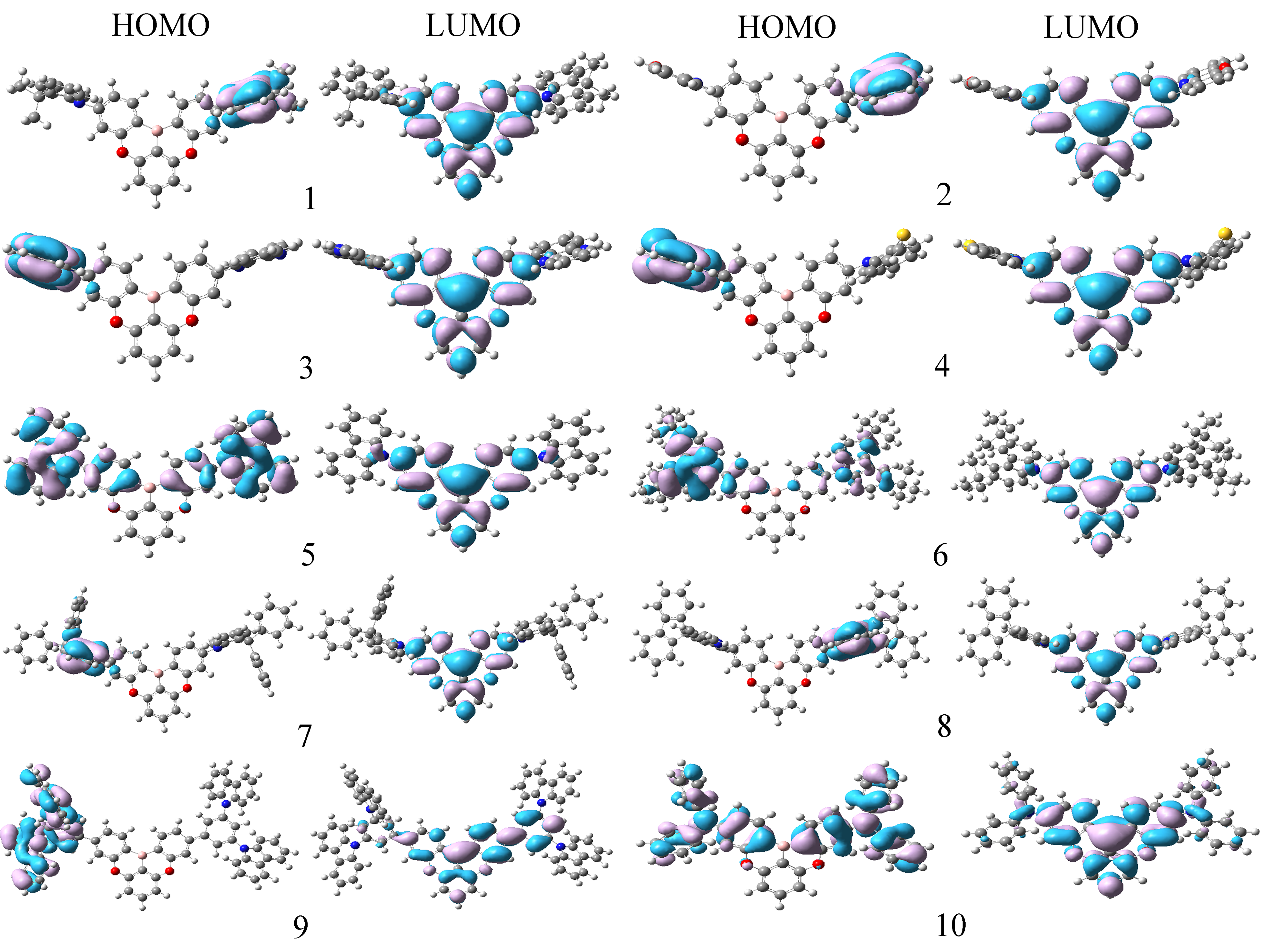
**

**Figure S3** The distributions of HOMOs and LUMOs in T_1_ states for the designed molecules.
